# Impact of a French social marketing campaign promoting pulse and whole grain consumption: results from a longitudinal cohort study

**DOI:** 10.3389/fnut.2023.1208824

**Published:** 2023-08-08

**Authors:** Philippine Fassier, Anaëlle Rabès, Pauline Ducrot, Anne-Juliette Serry

**Affiliations:** Department of Prevention and Health Promotion, Santé Publique France, The National Public Health Agency, Saint-Maurice, France

**Keywords:** diet, whole grains, dietary fiber, social marketing, knowledge, attitudes, practice

## Abstract

**Background:**

Despite the health benefits, fiber intake is insufficient among French adults. To promote the consumption of pulses and whole grains, defined as priority food groups because they are rich in fiber, readily available, and affordable, the French National Public Health Agency implemented a social marketing campaign in 2019 to improve knowledge, self-efficacy, and consumption of pulses and whole grains.

**Objective:**

To evaluate the short-and long-term effects of this social marketing campaign.

**Methods:**

A 8-month prospective study was conducted on the Internet. A sample of 4,001 French adults was interviewed before the social media campaign (T0), immediately afterwards (T1), and after 8 months (T2). Analysis was performed on 2,422 adults responding at T1 and T2. Outcomes associated with campaign recall were investigated by mixed models with random effects using generalized estimating equations.

**Results:**

Overall, 59.5% of subjects recalled the campaign. In multivariable-adjusted analyses, no significant difference was found in terms of knowledge, self-efficacy, and consumption of pulses and whole grains between those recalling and not recalling the campaign (value of *p* > 0.05). When the analyses were stratified by educational level (p-interaction<0.10), a significant positive association was found between campaign recall and variation of knowledge about whole grain fiber content in subjects with lower educational level (value of *p* = 0.002 at T1 and value of *p* = 0.008 at T2). For small consumers of pulses, subjects recalling the campaign improved both their knowledge (OR [95%CI] = 1.47 [1.14–1.90], value of *p* = 0.003 at T2) and consumption of pulses (1.44 [1.14–1.86], value of *p* = 0.002 at T1, 1.50 [1.16–1.94], value of *p* = 0.002 at T2).

**Conclusion:**

This first French social marketing campaign promoting pulses and whole grains had a positive impact on specific subgroups of particular interest in terms of public health (i.e., people with low educational level and small consumers of pulses). These results will allow us to improve the communication materials used for the second edition of this social marketing campaign.

## 1. Introduction

Given the well established relationship between dietary intakes and the heath (1–4), food-based dietary guidelines (FBDG) are established in many countries to help consumers make healthy dietary choices and adopt a healthy lifestyle, thus allowing them to achieve a satisfactory nutritional state. FBDG have been adapted to specific nutritional, geographical, economic, and cultural conditions (5).

In France, FBDG were issued for the first time in 2001 when the Ministry of Health initiated the National Nutrition and Health Program. In 2019, Santé publique France, the National Public Health Agency, updated and published new French FBDG for adults (6). These recommendations take into account the evolution of scientific research regarding the association between nutrition and health as well as the knowledge, beliefs, and dietary consumptions of the French population (6–8). The new guidelines notably include specific recommendations for the consumption of whole grains and pulses, the reduction of meat consumption (excluding poultry), and the consumption of local and seasonal foods to limit the environmental impact.^1^

The most recent representative survey conducted in France in 2014–2016 reported the poor consumption of pulses and whole grains despite their high fiber content (9). Indeed, 60% of French adults do not include whole grains in their diet, even though dietary guidelines recommend their consumption at least once a day, and more than 85% do not follow the recommendation to eat pulses at least twice a week. However, the current scientific evidence indicates that eating foods containing fiber is associated with a reduction in some chronic diseases (2, 10–12), including inflammatory diseases, cancers, cardiovascular diseases or type 2 diabetes, and indirectly reduces the risk of overweight and obesity (13).

Given the non-compliance of French adults with the fiber-related guidelines despite the health benefits (2, 10–13), guidelines to promote pulse and whole grain consumption were defined as a priority to be promoted, because these food groups are naturally rich in fiber, affordable, and easily available in food stores (14).

To promote the new French FBDG, and in particular the consumption of pulses and whole grains, Santé publique France implemented a social marketing campaign in 2019. The food literacy conceptual model was used to design the campaign (15) and, more specifically, the intrinsic component of the model that includes food skills, food and nutrition knowledge, self-efficacy, and confidence (including the self-perceived ability to apply nutritional, food, and cooking skills and the positive attitude toward healthy food) as determinants of behavior change. Women aged 25–49 years were the main target of this campaign, because women usually prepare meals, while women of low socio-professional status and with low educational level were particularly targeted given that they are specifically at risk of an unhealthy diet (9).

The main aims of this social marketing campaign were (1) to increase knowledge about the high fiber content of pulses and whole grains, (2) to improve self-efficacy by addressing perceived barriers (ease and time required to prepare these foods, pleasure of eating them, etc.), and (3) to encourage a healthier diet with more pulses and whole grains. The objectives of this study were therefore to evaluate the impact of the campaign on variations in (1) people’s knowledge, (2) self-efficacy (ease of preparation and pleasure of eating), and (3) consumption of pulses and whole grains between before and after campaign.

## 2. Materials and methods

### 2.1. French media campaign “Commencez par”

A new national social marketing campaign was launched on October 1, 2019, by Santé publique France. The 1-month campaign known as “Commencez par” (in English “Start with”) included several communication tools with different objectives that were disseminated throughout the entire campaign.

#### 2.1.1. One 30-s TV spot

This television advertisement aimed to reinforce self-efficacy by showing how to eat in healthier way without completely changing one’s diet or giving up one’s favorite food.

#### 2.1.2. One 15-s TV spot and one Internet advertising banner on pulses

These advertisements aimed to make people aware of the high fiber content of pulses and to encourage them to eat pulses more often by showing how they can be easily integrated into a dish that they usually eat.

The fiber content of whole grains and pulses is highlighted following the results of a qualitative study aimed at designing the new French FBDG (16). According to this study, knowledge about the high fiber content of pulses and whole grains can motivate French adults to consume these food groups.

#### 2.1.3. One 15-s TV spot and one Internet advertising banner on whole grain pasta

These advertisements aimed to make people aware of the high fiber content of whole grain pasta and to encourage them to eat it by showing how it can be used in their favorite pasta recipe.

#### 2.1.4. Two 45-s videos broadcast on Facebook and in the waiting rooms of health professionals

The two videos aimed to address some of the barriers to pulse and whole grain consumption, which were identified in a previous qualitative and quantitative study carried out to help formulate the new FBGD (16) (i.e., not knowing how to cook pulses and whole grains, believing that they are not tasty). The short videos gave some recipe ideas and showed that dishes cooked with whole grain foods and pulses can be tasty. They also demonstrated that cooking pulses and whole grain foods varies the diet and helps people to eat in a healthier way.

### 2.2. Study design

An 8-month prospective study was implemented from October 2019 to June 2020 to assess the effectiveness of the campaign. A sample of 4,001 participants aged 18 years and older was recruited in an access panel by a French market research company (BVA) in order to be interviewed via the Internet. The sample was selected according to quotas of sex, age, occupation, size of area of residence, and region, thus reflecting a representative sample of the French population. Respondents were first interviewed before the media campaign (T0). All were contacted again within 3 weeks of the end of the campaign (T1) and 8 months afterwards (T2).

### 2.3. Ethical consideration

Participation in this study was on a voluntary basis. Electronic informed consent was obtained from each participant before starting the questionnaire. Participants were given small incentives for participating and received compensation in the form of points, which could be accumulated and converted into different types of gifts. Personal data treatment was in accordance with French law n°78–17 of January 6, 1978, and European regulation n°2016/679, known as the General Data Protection Regulation.

### 2.4. Campaign recall assessment

Recalling the social marketing campaign was measured using the self-reported campaign recall at T1 by consecutively showing participants the different communication tools used throughout the campaign (three TV spots, two Internet advertising banners, and two videos broadcast on social networks and in the waiting rooms of health care professionals). For each one, participants were asked the following: “Here is an advertisement broadcast on television/Internet/waiting room of healthcare professional. Please indicate whether you remember seeing it.” Respondents were considered to recall the campaign if they reported seeing at least one of the campaign’s communication tools.

### 2.5. Knowledge about the high fiber content of pulses and whole grains

Participants’ knowledge about the fiber content of pulses and whole grains was measured by asking them to cite high fiber foods from a list of nine foods, including pulses and whole grains. If they selected pulses, they were considered as knowing that pulses are riches in fiber, and similarly if they selected whole grains, they were considered as knowing that whole grains are riches in fib.

### 2.6. Self-efficacy

Participants’ self-efficacy was first measured with regard to their ease of eating pulses and whole grains by asking them if it would be easy for them to eat more pulses and whole grains in the next 30 days. Answers proposed were: 1-very easy, 2- quite easy, 3- quite difficult disagree, 4- very difficult. For analyses, ease of eating pulses and whole grains was categorized in two classes: yes (= 1-very easy and 2- quite easy) or no (=3- quite difficult disagree and 4- very difficult).

Their self-efficacy was then measured with regard to their pleasure of eating pulses and whole grains by asking them if it would be pleasurable for them to eat more pulses and whole grains in the next 30 days. Answers proposed were: 1- strongly agree, 2- somewhat agree, 3 - somewhat disagree, 4 - strongly disagree For analyses, pleasure of eating pulses and whole grains was categorized in two classes: yes (= 1- strongly agree, 2- somewhat agree) and no (3- somewhat disagree, 4 - strongly disagree).

### 2.7. Consumption and pulses or whole grains

Pulse and whole grain consumption was measured by asking participants if they had consumed pulses or whole or semi-grain foods in the past 30 days. The proposed responses took into account the frequency of consumption as recommended by the FBGD: for pulses, (1) never, (2) less than once a week, (3) once a week, (4) twice a week, and (5) more than twice a week; and for whole grains (1) never, (2) less than once a week, (3) once a week, (4) several times a week but not every day, and (5) at least once a day. Pulse and whole grain consumption was then, respectively, defined as consuming pulses at least once a week (yes/no) and consuming whole grain cereals several times a week (yes/no). These cut-offs were lower than the corresponding guidelines due to the low consumption levels observed in the French population.

### 2.8. Covariates

Data on multiple potential confounding factors were collected at T0, including demographic, socioeconomic, and lifestyle-related factors. In particular, participants reported data regarding their sex (male, female), age (≤50, >50), area of residence (Paris, Paris suburbs, south, north, east, west, south-west), socio-professional category (high socio-professional category: managers, intellectual professions, self-employed; low socio-professional category: manual workers, middle-level professions; unemployed: retirees, students, other unemployed people), educational level (<high school diploma, high school diploma, >high school diploma), monthly income (<1,500, [1500–2,499], [2,500–3,499], ≥3,500€ per household, do not know or refused to answer), body mass index (BMI) (healthy weight < 25 kg/m2, overweight, or obese ≥25 kg/m2), and concerns about diet balance (yes, no).

### 2.9. Statistical analysis

#### 2.9.1. Study population

**Figure 1:**
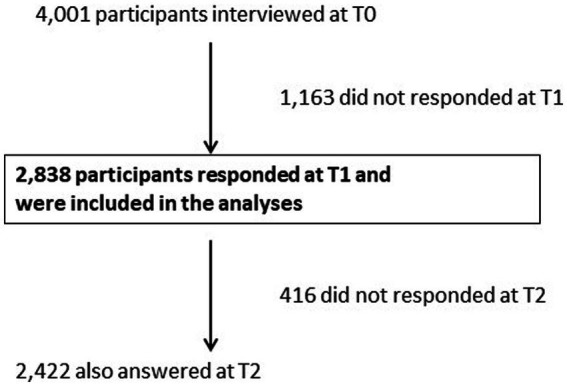
Flow chart.

Among the 4,001 participants interviewed at T0, 2,838 (71%) responded at T1 and were considered in the present analyses. Of them, 2,422 also answered at T2, which represents 60% of the population initially interviewed at T0 (Figure 1).

Data were weighted according to the French sociodemographic distribution in 2019 in terms of sex, age, socio-professional category, region, and size of area of residence.

#### 2.9.2. Descriptive analysis

Participants who recalled the campaign were compared to those who did not using Chi-square test. All demographic, socioeconomic, and lifestyle-related factors associated with recalling the campaign were included as confounding factors in multivariable-adjusted regressions.

#### 2.9.3. Association analysis

Mixed models were used with random effects using generalized estimating equations to evaluate the association between participants’ campaign recall and variation of consumption of pulses or whole grains and several determinants of consumption retained from the food literacy conceptual: knowledge and self-efficacy (ease and pleasure of eating) before and after the campaign. Outcomes of mixed models were the value of knowledge, self-efficacy, then consumption of pulses or whole grains at each different time; variables of interest were the participants’ campaign recall, the time and the interaction between participants’ campaign recall and time. *p*-values of the interaction are presented in the result section and in the figures. The mixed models were adjusted for sex, age, socio-professional categories, educational level, monthly income, BMI, and concerns about their diet balance.

Because the campaign targeted women aged 25–49 years of low socio-professional status, interactions with sex, socio-professional categories (low and high socio-professional categories, unemployed), and educational level (<high school diploma, high school diploma, or post-secondary graduate) were tested. If a statistically significant interaction was found, we performed stratified analysis.

#### 2.9.4. Sensitivity analysis

To evaluate if the campaign increased pulse or whole grain consumption and these determinants (knowledge and self-efficacy) among small consumers, we performed multivariable-adjusted logistic regressions for these specific participants to compare the pulse and whole grain consumption between participants who did and did not recall the campaign at T1 and T2. Small consumers were defined as those who declared consuming pulses less than once a week and whole grains once or less than once a week at T0. If a significant association was found, regarding consumption, the association with knowledge and self-efficacy was also investigated.

Analyses regarding the association between campaign recall and knowledge and self-efficacy were also made specifically among those with a poor level at baseline (those who had no knowledge and a poor self-efficacy).

All statistical analyses were performed using SAS Entreprise Guide V.7.1 (SAS Institute, Cary, North Carolina, USA). Statistical significance was set to *α* = 0.05 for all analyses except for the interaction analysis, which was set to *α* = 0.10.

## 3. Results

### 3.1. Study population characteristics

Individual characteristics of the weighted study population are presented in Table 1. Overall, 47.6% were women, 53.9% had less than 50 years old, 29.5% belonged to the upper socio professional category, and 51.8% had a healthy weight. In all, 59.5% (*n* = 1,689) of subjects recalled the campaign. More specifically, 57% recalled the TV spots, 23% recalled the Internet advertising banners, and 14% recalled the video broadcast on social networks and in the waiting room of health care professionals. Subjects who recalled the campaign were more likely to be women (value of *p* = 0.0001), older (value of *p* = 0.04), and with a low socio-professional category and low educational level (value of *p* = 0.007 and value of *p* = 0.003, respectively). They were also more likely to be overweight or obese (value of *p* = 0.0003) and to be concerned about their diet (value of *p* = 0.002).

**Table 1:** Characteristics of the weighted study population^1^ according to campaign recall (*n* = 2,838).

	All	Campaign recall		*p*-value ^1^
		Yes (*n* = 1,689)	No (*n* = 1,149)	
Sex, %				0.0001
Male	47.6	44.6	52.0	
Female	52.4	55.4	48.0	
Age (years), %				0.04
≤50	53.9	52.4	56.2	
>50	46.1	47.6	43.8	
Region, %				0.6
Paris/Paris surroundings	35.3	36.1	34.1	
South	25.0	25.1	24.9	
North/East	14.7	14.6	14.9	
West/South-west	25.0	24.3	26.1	
Socio-professional categories, %				0.007
Manager, intellectual and middle-level professions, and self-employed	29.5	27.4	32.6	
Manual workers	33.2	34.9	30.8	
Unemployed, retired, students	37.3	37.8	36.6	
Educational level, %				0.003
<High-school diploma	28.2	30.3	26.1	
High-school diploma	24.4	24.7	23.9	
>High-school diploma	47.4	45.0	51.0	
Monthly income (€ per household unit), %				0.5
<1500	16.1	16.9	15.0	
[1500–2499]	25.2	25.4	24.9	
[2500–3499]	21.9	21.7	22.2	
≥3500	22.9	21.9	24.2	
Do not know or refused to answer	13.9	14.1	13.6	
Body mass index, %				0.0003
Healthy weight (<25 kg/m^2^)	51.8	49.9	55.9	
Overweight or obese (≥25 kg/m^2^)	48.2	51.0	44.1	
Concerned about diet balance				0.002
Yes	76.0	78.0	73.0	
No	24.0	22.0	27.0	

1 *p*-value for the comparison between campaign recall using Chi-square test.

### 3.2. Knowledge about the fiber content of pulses and whole grains

**Figure 2:**
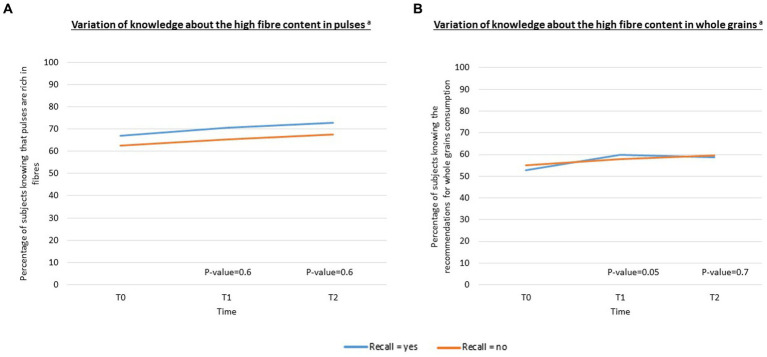
**(A,B)** Variations in the knowledge about the high fiber content of pulses and whole grains according to campaign recall (*N* = 2,838 at T0 and T1, *N* = 2,422 at T2). ^a^Adjusted for sex, age, socio-professional categories, educational level, monthly income, BMI and being concerned about their balance diet.

Among those who recalled the campaign, there was a 3.7% increase in subjects who knew that pulses are rich in fibers between T0 and T1 and 2.2% between T1 and T2 (Figure 2A). A similar trend was observed among those not recalling the campaign, with no significant difference in the variation between those who did and did not recall in multivariable-adjusted analyses (value of *p* = 0.6 at T1 and value of *p* = 0.6 at T2). When analyses were performed among those who had no knowledge regarding fibers contained in pulses at T0, results were no significant (data not shown).

Regarding the knowledge about the fiber content of whole grains, a significant difference in the variation was observed between participants who did and did not recall in multivariable-adjusted analyses at T1 (value of *p* = 0.05), although this association was non-significant at T2 (value of *p* = 0.7) (Figure 2B). When analyses were performed among those who had no knowledge regarding fibers contained in whole grains at T0, results were no significant (data not shown).

**Figure 3:**
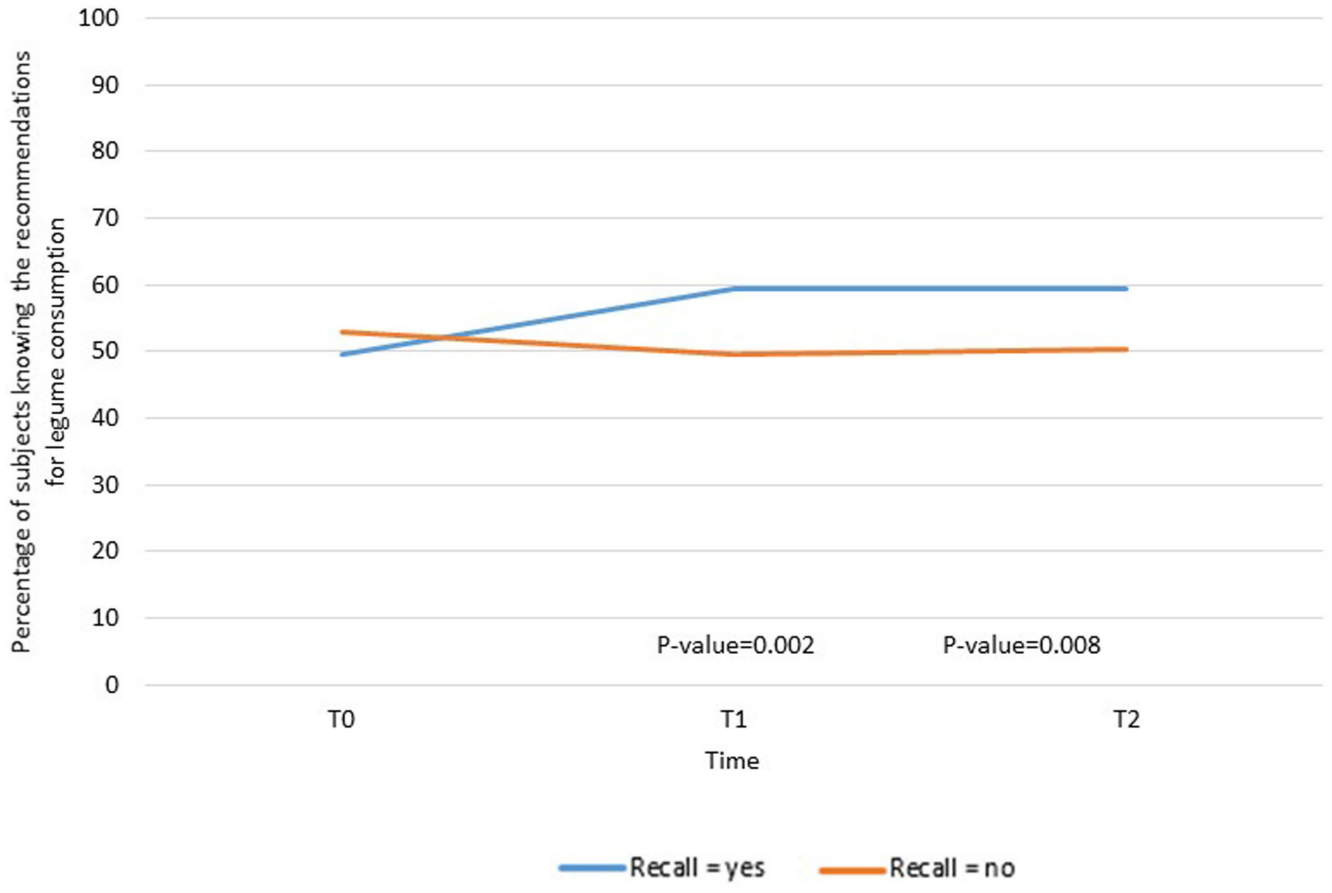
Variations in the knowledge about the high fiber content of whole grains according to campaign recall among subjects with low educational level (*N* = 799 at T0 and T1, *N* = 670 at T2). ^a^Adjusted for sex, age, socio-professional categories, monthly income, BMI and being concerned about their balance diet.

An interaction with educational level was found (value of *p* = 0.07 at T1 and value of *p* = 0.08 at T2, data not shown). Analyses were then stratified by educational level, and a significant positive association between campaign recall and knowledge of whole grain fiber content was found among those with lower educational level (value of *p* = 0.002 at T1 and value of *p* = 0.008 at T2) (Figure 3).

### 3.3. Self-efficacy about pulse and whole grain consumption

Regarding their ease of eating pulses and whole grains, no significant difference in the variation was observed between subjects who did and did not recall the campaign in multivariable-adjusted analyses (value of *p* = 0.6 at T1 and value of *p* = 0.2 at T2 for eating pulses and value of *p* = 0.1 at T1 and value of *p* = 0.1 at T2 for eating whole grains) (Appendix 1). When analyses were performed among those declaring it is not easy to eat pulses or whole grains at T0, results remained no significant (data not shown).

Moreover, there were no significant differences between subjects who did and did not recall the campaign concerning their pleasure of eating pulses and whole grains in multivariable-adjusted analyses (value of *p* = 0.4 at T1 and value of *p* = 0.6 at T2 for eating pulses and value of *p* = 0.4 at T1 and value of *p* = 0.4 at T2 for eating whole grains) (Appendix 2). When analyses were performed among those who reported no pleasure to eat pulses or whole grains at T0, a positive association was found regarding pleasure to eat whole grain at T1 (OR [95%CI] =1.78 [1.22–2.60]) but not at T2 (0.87 [0.58–1.31]) (data not shown).

### 3.4. Pulse and whole grain consumption

**Figure 4:**
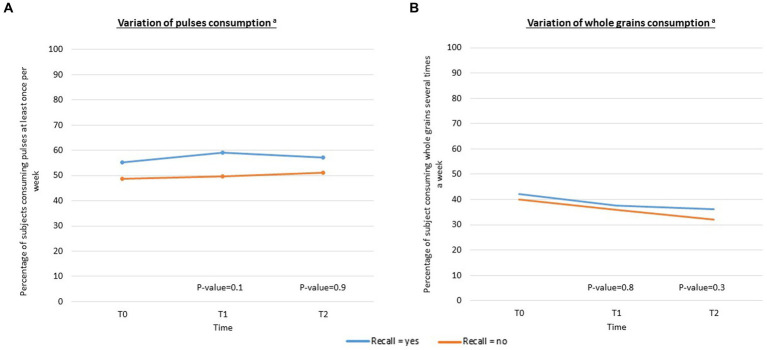
**(A,B)** Variations in pulse and whole grain consumption according to campaign recall (*N* = 2,838 at T0 and T1, *N* = 2,422 at T2). ^a^Adjusted for sex, age, socio-professional categories, educational level, monthly income, BMI and being concerned about their balance diet.

Among participants who recalled the campaign, there was a 3.7% increase in those who consumed pulses at least once a week between T0 and T1 but a 1.8% decrease between T1 and T2 (Figure 4A). Among participants who did not recall the campaign, there was a 0.8% increase between T0 and T1 and a 0.5% increase between T1 and T2. In multivariable-adjusted analyses, the difference in the variation between those who did and did not recall the campaign was non-significant (value of *p* = 0.1 at T1 and value of *p* = 0.9 at T2).

Among participants who recalled the campaign, the percentage of subjects consuming whole grains several times a week decreased by 4.7% between T0 and T1 and by 1.4% between T1 and T2 (Figure 4B). Among participants who did not recall the campaign, there was a 4.0% decrease between T0 and T1 and a 3.9% decrease between T1 and T2. In multivariable-adjusted analyses, the difference in the variation between those who did and did not recall was non-significant (value of *p* = 0.8 at T1 and value of *p* = 0.3 at T2).

### 3.5. Sensitivity analysis

Regarding the small consumers of pulses:

- Those who recalled the campaign were more likely to consume pulses at least once a week at T1 (OR [95%CI] =1.44 [1.14–1.86]) and T2 (1.50 [1.16–1.94]) (Table 2) compared to their counterparts who did not recall.
- Those recalling the campaign were more likely to know that pulses are rich in fibers at T2 (1.48 [1.15–1.91]) (non-significant association at T1) (Table 2). No association was found in terms of their self-efficacy (ease and pleasure) of eating pulses (data not shown).
- When the analysis of the association between campaign recall and pulse consumption was stratified by knowledge about the fiber content of pulses, the association remained statistically significant only among those who knew about the high fiber content of pulses (1.56 [1.16–2.08] at T1 and 1.66 [1.21–2.28] at T2) (data not shown).

**Table 2:** Association between pulse and whole grain consumption and campaign recall among small consumers (*N* = 1343 for small consumers of pulses, *N* = 1667 for small consumers of whole grains).

	Pulse consumption at least once a week				Whole grain consumption several times a week			
	T1		T2		T1		T2	
	OR [95%CI]	*p*-value^1^	OR [95%CI]	*p*-value^1^	OR [95%CI]	*p*-value^1^	OR [95%CI]	*p*-value^1^
Campaign recall								
No	1 (ref)	0.002	1	0.002	1	0.4	1	0.09
Yes	1.44 [1.14–1.86]		1.50 [1.16–1.94]		1.10 [0.87–1.40]		1.25 [0.96–1.61]	
	Knowledge about the high fiber content of pulses							
	T1		T2					
	OR [95%CI]	*p*-value^1^	OR [95%CI]	*p*-value^1^				
Campaign recall								
No	1 (ref)	0.2	1 (ref)	0.003				
Yes	1.17 [0.93–1.48]		1.47 [1.14–1.90]					

1 Adjusted for sex, age, socio-professional category, educational level, monthly income, body mass index, and being concerned about their diet balance.

Regarding the small consumers of whole grains:

- There was no significant difference between those who did and did not recall the campaign regarding their whole grain consumption at T1 and T2 (value of *p* = 0.4 and value of *p* = 0.09, respectively) (Table 2).

## 4. Discussion

This longitudinal study conducted among 2,422 French adults provided interesting findings about the effectiveness of a French social marketing campaign to promote pulse and whole grain consumption. Almost 60% of subjects reported recalling the campaign, with a higher recall prevalence among people with a low socio-professional category and low educational level. Overall, this first broadcast of the campaign had limited effects on pulse and whole grain consumption as well as on the determinants studied here (i.e., knowledge, self-efficacy). However, among subjects with low educational level, a positive association was observed between campaign recall and variation of their knowledge about the fiber content of whole grains. Moreover, when focusing on the small consumers of pulses, those recalling the campaign improved their knowledge about the fiber content of pulses and consumed more pulses in the immediate aftermath of the campaign and 8 months later.

Social marketing is a method used to analyze, plan, execute, and evaluate programs to influence the behavior of the target audiences in order to improve their personal welfare or that of society (17). The “Eating for the Better” review (18) identified the success factors and potential barriers to social marketing for healthy eating behaviors based on 34 empirical studies. The observed healthy eating behaviors were eating fruit and vegetables, low-fat food, low-salt food, and choosing healthy foods. Of the 16 studies considered to be social marketing according to the criteria of Andreasen (17), all measured positive changes for at least two of the measured indicators such as fruit and vegetable intake or better knowledge. Diet-related social marketing campaigns or mass media campaigns generally aimed to increase fruit and vegetable consumption (19–21). However, to our knowledge, no study to date has evaluated the impact of a social marketing campaign targeting pulse or whole grain consumption. The CVD Prevention Through Policy review and the American Heart Association (22, 23) concluded that communication campaigns were effective in improving dietary behaviors, particularly for fruit and vegetable consumption and salt reduction, which were the main outcomes of these campaigns. However, some reviews (24, 25) found that although communication campaigns do raise awareness about dietary recommendations, they fail to translate the message into behavior changes. For the authors, social marketing campaigns should be repeated over a long period to achieve eating behavior changes. One possible explanation is that the environment in which individuals live includes various factors that continuously encourage unhealthy behaviors (26). All the reviews investigating the effectiveness of diet-based interventions, including social marketing campaigns, concur that too few studies evaluate these interventions, thus leading to a publication bias (18, 22, 24, 27).

Our results point to improved knowledge about the fiber content of whole grains among participants with low educational level who recalled the campaign, although this knowledge did not translate into eating behavior changes. In addition to improved knowledge, this social marketing campaign aimed to influence other barriers to the consumption of whole grains such as a lack of cooking skills or desire to eat them. The campaign could not address the cost issue, which is another important barrier to their consumption (28–30). Unfortunately, no variations in the perceived ease or pleasure of eating whole grains were observed, excepted among those who had no pleasure to eat whole grain at T0, for which a positive association with campaign recall was found at T1. One possible explanation is the low recall of the videos broadcast on social networks and in the waiting room of health professionals (14%), as they aimed to overcome these barriers.

Our results are in accordance with previous reviews showing that knowledge improvement alone is insufficient to improve eating behavior (24, 25). Regarding other countries, lessons can be learned from Denmark, where the consumption of whole grains increased significantly from an average of 36 g per day in 2007 to 63 g in 2014 (31). This improvement might be related to the implementation of the Danish Whole Grain Partnership (DWGP) in the mid-2000s, a public-private partnership aiming to boost whole grain consumption (32). The actions of this partnership included educational campaigns and events, the promotion of whole grains as a climate-positive food, the increased availability of whole grain products, and their promotion through front-of-pack labelling to allow consumers to easily identify whole grain foods (33).

Another interesting result of our study relates to the small consumers of pulses who increased their consumption of pulses. This increase was only observed among the small consumers for whom the campaign improved their knowledge about fiber richness, which could partly explain this result. This increase in consumption might be surprising, since the campaign had no impact on self-efficacy (namely, ease and pleasure of eating pulses), even though these determinants have been described in the literature as important barriers to pulse consumption (34, 35) and were thus taken into account when designing the campaign. Indeed, two recent French studies (36, 37) published during and after the campaign confirmed that the perceived difficulty of cooking pulses as well as taste preferences could be among the main obstacles to their consumption. As previously mentioned, one possible explanation for the absence of self-efficacy variations in our study may be the low recall of the video broadcast on social networks and in the waiting rooms of health professionals. To explain the increase in pulse consumption among small consumers, it is important to acknowledge that positive communication and actions around pulse consumption have been undertaken in France in the last few years. This notably occurred following some political initiatives such as the weekly meat-and fish-free meal in French school canteens and communication campaigns regarding the impact of consumption choices on the environment. Therefore, the “Commencez par” campaign initiated in this favorable context may have changed people’s perception of pulses (being difficult to cook, not tasty, etc.) and increased their consumption.

One major strength of this study was the prospective evaluation of pulse and whole grain consumption as well as their determinants, collected before and after the broadcast of the social marketing campaign with detailed information regarding potential confounding factors.

However, several limitations should be acknowledged. First, as the cohort involved volunteers, it included a potential selection bias, as volunteers are more often concerned about their health status and dietary behaviors. Their level of whole grain and pulse consumption was higher than in the general population, which limited their scope for improvement. Despite multiple adjustments regarding the measurement of associations with campaign recall, confounding bias cannot be completely eliminated. Furthermore, only 60% of respondents at T0 completed the questionnaires at T1 and T2. Despite performing post-stratification weighting, we can assume a potential attrition bias.

In conclusion, this first French social marketing campaign promoting pulses and whole grains highlighted some positive impacts for specific subgroups of particular interest in terms of public health. The campaign improved people’s knowledge about the fiber content of whole grains, although this did not change the level of consumption among people with low educational level. Nonetheless, this latter group is a priority target to reduce health inequalities. The campaign also increased pulse consumption among small consumers, probably by increasing their knowledge, which may have been bolstered by the favorable context to consume pulses with other structural initiatives undertaken in the same period such as the introduction of weekly meat-and fish-free meals in school canteens. However, these changes were observed among participants who were exposed to a single social marketing campaign, even though it is known that longer campaigns are more effective to change eating behaviors (25). These encouraging results highlight the interest of rebroadcasting the campaign while taking into account the lessons learned from this evaluation and adapting the communication materials for the second wave. Thus, the ease of preparing of whole grains and pulses and their tastiness were identified as key messages to be reinforced to encourage behavior change. Further evaluations will be needed to investigate the effects of the redesigned campaign after a second edition.

## Data availability statement

The raw data supporting the conclusions of this article will be made available by the authors, without undue reservation.

## Ethics statement

Ethical approval was not provided for this study on human participants because participation in this study was on a voluntary basis. Electronic informed consent was obtained from each participant before starting the questionnaire. Participants were given small incentives for participating and received compensation in the form of points, which could be accumulated and converted into different types of gifts. Personal data treatment was in accordance with French law n°78–17 of January 6, 1978, and European regulation n°2016/679, known as the General Data Protection Regulation. The patients/participants provided their written informed consent to participate in this study.

## Author contributions

A-JS and PD designed the research and conducted the research. AR and PF performed the statistical analysis and wrote the paper. A-JS, AR, PD, and PF analyzed the data, revised paper for important intellectual content, and read and approved final manuscript. PF had primary responsibility for final content. All authors contributed to the article and approved the submitted version.

## Conflict of interest

The authors declare that the research was conducted in the absence of any commercial or financial relationships that could be construed as a potential conflict of interest.

## Publisher’s note

All claims expressed in this article are solely those of the authors and do not necessarily represent those of their affiliated organizations, or those of the publisher, the editors and the reviewers. Any product that may be evaluated in this article, or claim that may be made by its manufacturer, is not guaranteed or endorsed by the publisher.

## Figures and Tables

**APPENDIX 1:**
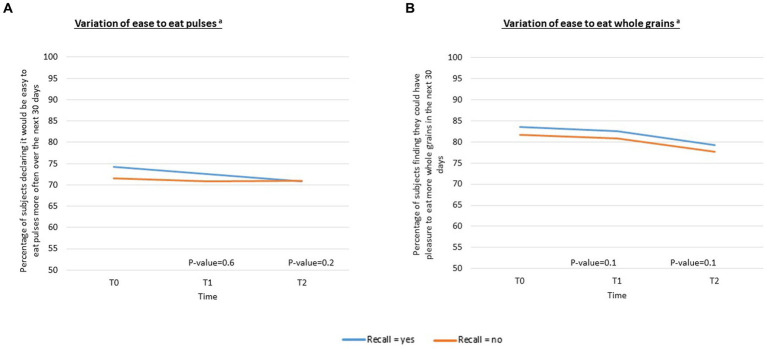
**(A,B)** Variations in the ease of eating pulses and whole grains according to campaign recall (*N* = 2838 at T0 and T1, *N* = 2422 at T2). ^a^Adjusted for sex, age, socio-professional categories, educational level, monthly income, BMI and being concerned about their balance diet.

**APPENDIX 2:**
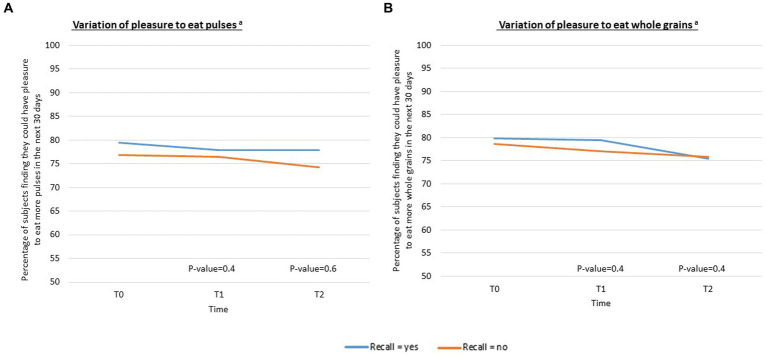
**(A,B)** Variations in the pleasure of eating pulses and whole grains according to campaign recall (*N* = 2838 at T0 and T1, *N* = 2422 at T2). ^a^Adjusted for sex, age, socio-professional categories, educational level, monthly income, BMI and being concerned about their balance diet.

## Footnotes

^1^https://www.mangerbouger.fr/l-essentiel/les-recommandations-sur-l-alimentation-l-activite-physique-et-la-sedentarite
